# High-Intensity Interval Training on Cardiorespiratory Fitness, Cognitive Function, and Functional Capacity in Adults with Stroke: A Systematic Review and Meta-Analysis

**DOI:** 10.3390/jcm15134977

**Published:** 2026-06-26

**Authors:** Javier Cano-Sánchez, Raquel Fábrega-Cuadros, Yulieth Rivas-Campo, Camila Perafan-Grajales, María del Carmen Carcelén-Fraile, Juan Miguel Muñoz-Perete

**Affiliations:** 1 Department of Health Sciences, Faculty of Health Sciences, University of Jaén, 23007 Jaen, Spain; jcs00033@red.ujaen.es (J.C.-S.); jmmunoz@ujaen.es (J.M.M.-P.); 2 Faculty of Human and Social Sciences, University of San Buenaventura-Cali, Santiago de Cali 760016, Colombia; 3 Western Clinical Research and Education Group (GIEDCO Western Clinic), Santiago de Cali 760046, Colombia; 4 Department of Educational Sciences, Faculty of Social Sciences, University of Atlántico Medio, 35017 Las Palmas de Gran Canaria, Spain

**Keywords:** stroke, high-intensity interval training, HIIT, cardiorespiratory fitness, functional capacity, cognitive function, neurorehabilitation, gait performance, meta-analysis

## Abstract

**Background/Objectives:** Stroke is a leading cause of long-term disability worldwide and is frequently associated with reduced cardiorespiratory fitness, impaired functional capacity, and cognitive decline. High-intensity interval training (HIIT) has emerged as a promising rehabilitation strategy; however, its effects across multiple domains remain unclear. This systematic review and meta-analysis aimed to evaluate the effects of HIIT on cardiorespiratory fitness, cognitive function, and functional capacity in adults with stroke. **Methods:** A systematic search was conducted in PubMed, Scopus, CINAHL, and Web of Science up to April 2026. Randomized controlled trials involving HIIT interventions in adults with stroke were included. Outcomes of interest were cardiorespiratory fitness (VO_2_peak), functional capacity (gait speed, walking distance, mobility, and balance), and cognitive function. Pooled effect sizes were calculated using a random-effects model. Methodological quality was assessed using the PEDro scale, and risk of bias was evaluated with the Cochrane RoB-2 tool. **Results:** In total, 17 studies (n = 809 participants) were included, with 12 contributing to the meta-analysis. HIIT significantly improved cardiorespiratory fitness (SMD = −0.849; *p* = 0.005), gait speed (SMD = 0.693; *p* = 0.014), walking distance (SMD = 0.604; *p* < 0.001), functional mobility (SMD = −0.711; *p* = 0.027), balance (SMD = 2.447; *p* = 0.002), and cognitive function (SMD = 1.741; *p* < 0.001). However, substantial heterogeneity was observed across most outcomes. **Conclusions:** HIIT appears to be an effective intervention for improving cardiorespiratory fitness, functional capacity, and cognitive performance in individuals with stroke. Nevertheless, the variability across studies suggests that its effectiveness is context-dependent. Further research is needed to standardize protocols and determine optimal implementation strategies.

## 1. Introduction

Stroke is one of the leading causes of mortality and long-term disability worldwide and represents a substantial burden for healthcare systems, patients, and caregivers [1]. According to the Global Burden of Disease study, stroke remains the second leading cause of death globally and one of the primary contributors to disability-adjusted life years (DALYs), affecting millions of individuals each year [2]. Recent epidemiological reports estimate that more than 12 million new stroke cases occur annually worldwide, with approximately 6.5 million stroke-related deaths [3]. Improvements in emergency care, thrombolytic therapies, and acute stroke management have significantly increased survival rates; however, this has also led to a growing population of stroke survivors living with chronic physical, cognitive, and psychosocial impairments [4].

Stroke is generally classified into ischemic and hemorrhagic subtypes, with ischemic stroke accounting for nearly 85% of all cases [5]. Regardless of etiology, stroke frequently results in neurological damage that compromises motor, sensory, cognitive, and cardiovascular function [6]. The extent of disability depends on lesion location, severity, rehabilitation access, and pre-existing health conditions [7]. Common post-stroke impairments include hemiparesis, reduced balance, gait disturbances, fatigue, cognitive decline, reduced aerobic capacity, and decreased ability to perform activities of daily living independently. These limitations often contribute to sedentary behavior, social isolation, recurrent cardiovascular events, and diminished quality of life [8].

One of the most frequently overlooked consequences of stroke is the marked decline in cardiorespiratory fitness [9]. Individuals who survive stroke typically demonstrate significantly lower peak oxygen consumption (VO_2_peak) compared with age-matched healthy adults, often reaching levels below those required for independent living [10]. This reduction may be explained by physical inactivity, impaired autonomic regulation, skeletal muscle deconditioning, reduced mitochondrial efficiency, and cardiovascular dysfunction following the cerebrovascular event [11]. Lower cardiorespiratory fitness has been strongly associated with poorer walking performance, reduced participation in rehabilitation programs, increased cardiovascular risk, and higher mortality rates [12].

In addition to physical limitations, cognitive impairment is highly prevalent after stroke and affects approximately one-third of survivors [13]. Post-stroke cognitive dysfunction may involve deficits in executive function, memory, attention, language processing, and information processing speed [14]. These impairments may appear immediately after the cerebrovascular event or emerge progressively over time due to vascular degeneration and secondary neurological complications. Cognitive deficits significantly influence functional independence, adherence to rehabilitation programs, return to work, and overall long-term prognosis [15].

Functional capacity is another critical outcome in stroke rehabilitation. Functional limitations often manifest through reduced walking speed, impaired balance, lower muscular endurance, and difficulties performing activities such as climbing stairs, transferring positions, and completing daily tasks [16]. Restoration of functional independence is considered one of the principal goals of post-stroke rehabilitation because it directly influences quality of life and reduces caregiver burden [17].

Exercise-based rehabilitation has been widely recognized as a fundamental component of stroke recovery [18]. Current clinical guidelines recommend aerobic exercise, resistance training, balance exercises, and task-specific rehabilitation strategies to improve mobility, cardiovascular health, and physical function [19]. Among aerobic exercise modalities, moderate-intensity continuous training (MICT) has traditionally been the most commonly prescribed intervention due to its perceived safety and feasibility. Numerous studies have shown that MICT can improve walking performance, aerobic fitness, and cardiovascular health in stroke survivors [20,21].

However, despite these benefits, MICT may require longer training sessions and may not always generate sufficient physiological stimulus to optimize recovery [22]. In recent years, high-intensity interval training (HIIT) has emerged as an alternative exercise strategy characterized by repeated bouts of vigorous exercise interspersed with periods of active or passive recovery. HIIT protocols are often shorter in duration while producing substantial cardiovascular and metabolic adaptations [23].

The physiological rationale supporting HIIT in stroke rehabilitation is particularly compelling [24]. High-intensity exercise may stimulate greater improvements in stroke volume, endothelial function, mitochondrial biogenesis, insulin sensitivity, and skeletal muscle oxidative capacity compared with moderate-intensity training [25]. Furthermore, HIIT may enhance neuroplasticity through increased cerebral blood flow, upregulation of brain-derived neurotrophic factor (BDNF), improved angiogenesis, and facilitation of cortical reorganization. These mechanisms suggest that HIIT may positively influence both physical recovery and cognitive performance after stroke [26].

HIIT has already demonstrated effectiveness in several clinical populations, including patients with cardiovascular disease, obesity, type 2 diabetes, Parkinson’s disease, and multiple sclerosis [27]. In these populations, HIIT has been associated with improvements in aerobic fitness, vascular health, metabolic regulation, fatigue management, and cognitive outcomes. Given the overlap in cardiovascular and neurological impairments between these conditions and stroke, HIIT may represent a promising rehabilitation strategy for stroke survivors [25].

Despite growing interest in this area, studies investigating HIIT after stroke have reported heterogeneous findings. Variations in exercise protocols, session duration, training frequency, participant age, stroke severity, and outcome measures make it difficult to draw definitive conclusions. While some randomized controlled trials have reported significant improvements in VO_2_peak, walking capacity, and cognitive performance, others have shown limited or non-significant effects [28].

Although previous systematic reviews have examined exercise interventions in stroke populations, few have specifically focused on the combined effects of HIIT on cardiorespiratory fitness, cognitive function, and functional capacity [18,29,30]. Moreover, recent randomized controlled trials have expanded the available evidence base, highlighting the need for an updated synthesis of current findings.

Therefore, the purpose of this systematic review and meta-analysis was to evaluate the effects of high-intensity interval training on cardiorespiratory fitness, cognitive function, and functional capacity in adults with stroke. The findings of this review may provide clinicians, rehabilitation specialists, and policymakers with evidence-based guidance regarding the implementation of HIIT in stroke rehabilitation programs.

## 2. Materials and Methods

This review was conducted following the 2020 PRISMA statement guidelines [31] (Table S1) and adhered to a pre-registered protocol in PROSPERO (CRD420261374766). In addition, the methodological framework was guided by the recommendations outlined in the Cochrane Handbook for Systematic Reviews of Interventions [32].

### 2.1. Sources of Information

A comprehensive search of the literature was performed in April 2026 across the PubMed, Scopus, CINAHL, and Web of Science (WOS) databases.

### 2.2. Search Strategy

Multiple search terms were utilized in the following search string: (stroke OR ischemic stroke OR hemorrhagic stroke OR cerebrovascular accident OR acute cerebrovascular accident) AND (HIIT OR high-intensity interval training OR interval training OR intermittent training OR high-intensity exercise OR vigorous exercise OR aerobic interval training OR sprint interval training). No language restrictions were applied during the literature search. Studies published in any language were considered eligible for inclusion, provided that sufficient information was available for screening and data extraction.

### 2.3. Inclusion Criteria

The articles selected had to meet the following inclusion criteria: (i) the studies had to be randomized controlled trials (RCTs); (ii) the intervention had to involve high-intensity interval training (HIIT) or related modalities, including interval training, intermittent training, or other forms of vigorous or high-intensity aerobic exercise; (iii) participants had to be adults diagnosed with stroke, including ischemic stroke, hemorrhagic stroke, or acute cerebrovascular accident; and (iv) the studies had to specifically evaluate the effects of these exercise interventions on individuals with a confirmed diagnosis of cerebrovascular accident.

### 2.4. Exclusion Criteria

Articles were excluded if they met any of these criteria: (i) studies without a non-intervention or control comparison group; (ii) studies that did not assess outcomes related to the effects of HIIT or related high-intensity exercise interventions; (iii) the presence of participants without a confirmed diagnosis of stroke, or mixed populations without separate stroke-specific data; and (iv) interventions that did not include structured high-intensity interval training or related forms of vigorous exercise (for example, pharmacological, nutritional, or rehabilitation interventions without a HIIT component).

### 2.5. Study Selection Process

The process of selecting studies began with the removal of duplicate records and articles lacking available abstracts. Titles and abstracts were carefully examined to exclude those that did not align with the predefined eligibility criteria. The remaining articles were then assessed in full text to determine their appropriateness for inclusion in the meta-analysis. To maintain objectivity and minimize bias, two authors (R.F.-C. and J.M.M.-P.) independently conducted the selection process. Any disagreements regarding study eligibility were resolved through consultation with a third author (Y.R.-C.), who helped reach a consensus. This meticulous process ensured that all included studies were pertinent and met the established criteria.

### 2.6. Data Extraction

Two authors (J.C.-S. and M.d.C.C.-F.) independently extracted data from all included studies using a standardized data extraction form. The following information was collected: author and year of publication, country, sample characteristics, stroke type, participant age and sex, intervention and control group characteristics, HIIT protocol parameters (frequency, duration, intensity, and total intervention period), outcome measures, and main findings. When necessary, disagreements regarding data extraction were resolved through discussion and consultation with a third author (J.M.M.-P.) until consensus was reached. The extracted data were subsequently used for the qualitative synthesis and, where appropriate, for the quantitative meta-analysis.

### 2.7. Assessment of Methodological Quality

Methodological quality was assessed using the PEDro scale [33], which consists of an 11-item checklist. The highest possible score is 10 points, since the first item related to eligibility criteria is not included in the total score. Each criterion is rated dichotomously as “Yes” (1 point) or “No” (0 points). Study quality was classified according to the following categories: scores from 0 to 3 were considered “Poor,” 4 to 5 “Fair,” 6 to 8 “Good,” and scores above 9 “Excellent” [34].

In addition, risk of bias was evaluated using the Cochrane RoB-2 tool, specifically developed for randomized studies, particularly randomized clinical trials. This instrument assesses five principal domains and categorizes the overall risk of bias for each study as low, high, or unclear [35].

### 2.8. Analytic Decisions for Meta-Analysis

Meta-analyses were conducted when at least two studies reported comparable outcomes and provided sufficient quantitative data for pooling. The primary outcomes included cardiorespiratory fitness (e.g., VO_2_peak, VO_2_max), cognitive function (e.g., executive function, memory, and attention), and functional capacity (e.g., walking performance, functional mobility, and activities of daily living).

For continuous outcomes, pooled effect sizes were calculated using mean differences (MDs) when studies used the same measurement scale. When different instruments were used to evaluate similar constructs, standardized mean differences (SMDs) with 95% confidence intervals (CIs) were calculated. Post-intervention means and standard deviations were extracted from both intervention and control groups. When studies reported change-from-baseline values instead of post-intervention values, these were also included according to Cochrane recommendations.

Considering the expected methodological and clinical heterogeneity across studies—including variations in HIIT protocols, intervention duration, participant characteristics, and outcome assessment tools—a random-effects model was applied for all analyses. Statistical heterogeneity was assessed using the I^2^ statistic, with values interpreted as low (<25%), moderate (25–50%), substantial (50–75%), and considerable (>75%) heterogeneity. Cochran’s Q test was also examined, with statistical significance set at *p* < 0.10 due to the low statistical power of this test when few studies are included. Where sufficient studies were available, subgroup analyses were planned according to stroke type, intervention characteristics, and outcome domains. Sensitivity analyses were performed by excluding studies with high risk of bias to evaluate the robustness of the findings. Publication bias was assessed through visual inspection of funnel plots when at least ten studies were available for a specific outcome. Statistical significance for pooled analyses was established at *p* < 0.05. All statistical analyses were performed using Comprehensive Meta-Analysis software (CMA), version 3.0 (Biostat, Englewood, NJ, USA).

## 3. Results

### 3.1. Study Characteristics

**Figure 1:**
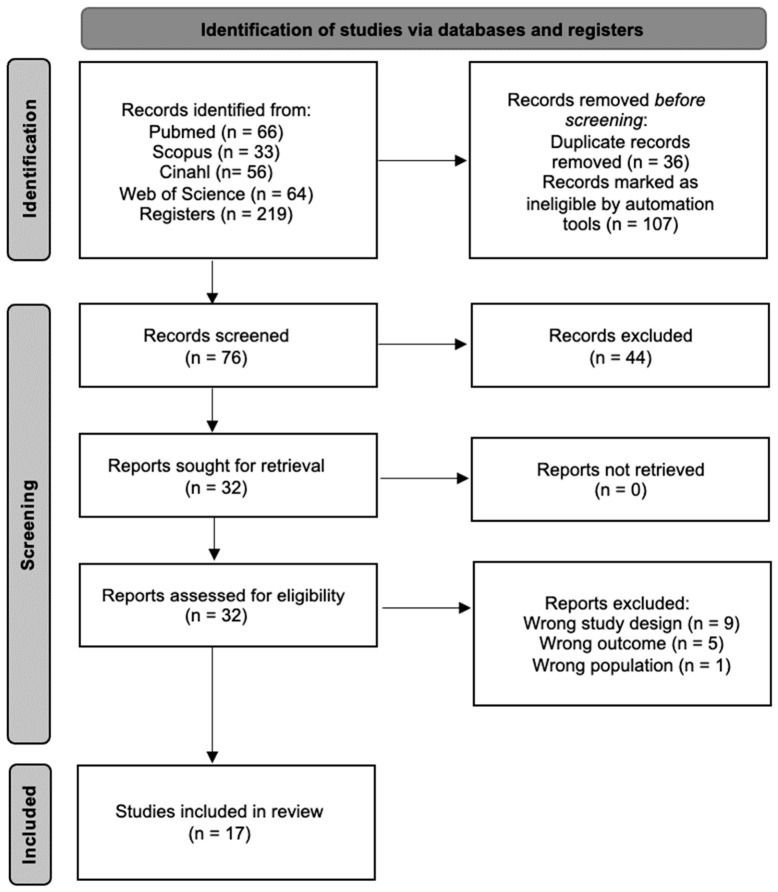
Study selection process flow chart.

The initial search conducted across several databases yielded 219 records. This set was subsequently refined by limiting the results to specific document types (articles and randomized clinical trials), applying keyword filters to titles and abstracts, and removing duplicate entries, resulting in 76 unique studies. Following this, a screening of titles and abstracts was performed, reducing the pool to 32 studies considered potentially eligible for qualitative assessment. Ultimately, 17 articles [36,37,38,39,40,41,42,43,44,45,46,47,48,49,50,51,52] satisfied the inclusion criteria and were included in the systematic review. Of these, 12 studies provided sufficient quantitative data and were incorporated into the meta-analysis, whereas five studies were included only in the qualitative synthesis. The remaining 15 articles were excluded after full-text assessment. The full selection process is illustrated in Figure 1.

### 3.2. Methodological Quality and Risk of Bias

The methodological quality of the studies included was assessed using the PEDro scale, with scores obtained from the PEDro website. All articles were rated as “Good”, except for one study, which was classified as “Excellent”. To maintain objectivity and consistency in the evaluation, two independent reviewers scored the studies based on the PEDro scale. In cases of disagreement, a conflict resolution procedure was followed. The reviewers discussed the differences, and if they could not reach a consensus, a third reviewer was involved. This approach ensured that the study grading was as accurate and reliable as possible. A comprehensive assessment of the methodological quality can be found in Table 1.

The risk of bias was assessed using the Cochrane RoB-2 tool, with each study categorized as having a low, unclear, or high risk of bias. Of the 17 articles included, 8 were rated as low risk, 1 as unclear risk, and the remaining 8 were also classified as either low or unclear risk. A detailed breakdown of the evaluation by domain is provided in Table 2.

### 3.3. Characteristics of the Included Studies

All studies included in this systematic review and meta-analysis were randomized controlled trials conducted in Benin [36], the United States [37,38,50], South Korea [39,43,44,48], Norway [40,41], Taiwan [42], Denmark [45], and Canada [46,47,49,51,52]. In total, 809 participants were involved, with 395 assigned to the control group and 414 to the intervention group, which focused on physical activity. A higher proportion of males was observed among the overall participants included in the review. The mean age of participants was 60.72 years (Table 3).

### 3.4. Outcome Measures

Of the 17 studies included in this systematic review, 12 were incorporated into the meta-analysis, while 5 studies [36,49,50,51,52] were excluded from the quantitative synthesis because it did not assess the primary outcomes of interest. The variables examined in this systematic review were cardiorespiratory fitness, functional capacity and cognitive function. Functional capacity encompassed several domains, including gait speed, walking distance, functional mobility, and balance.

Cardiorespiratory fitness was assessed in eight studies [37,38,39,40,42,43,46,48], exclusively using VO_2_peak obtained through graded exercise tests. Functional capacity was the most frequently assessed outcome, evaluated across multiple studies [37,38,39,41,43,44,46,47,48] using a variety of instruments. These included gait speed assessed by the 10-Meter Walk Test (10MWT), walking distance measured by the Six-Minute Walk Test (6MWT), functional mobility evaluated with the Timed Up and Go (TUG) test, and balance performance assessed using the Berg Balance Scale (BBS). Cognitive function was evaluated in three studies [41,45,46], most commonly using the Montreal Cognitive Assessment (MoCA) to assess global cognitive performance.

### 3.5. Meta-Analysis of Cardiorespiratory Fitness

**Figure 2:**
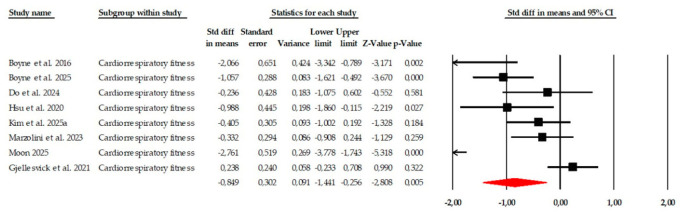
Forest plot of the effects of high-intensity interval training on cardiorespiratory fitness (VO_2_peak) in individuals with stroke. Black squares represent the effect estimates of individual studies, horizontal black lines indicate the corresponding 95% confidence intervals, and the red diamond represents the pooled effect estimate. Arrows indicate the direction of the effect.

Seven randomized controlled trials evaluating cardiorespiratory fitness were included in the meta-analysis, with VO_2_peak as the primary outcome measure. The pooled random-effects model demonstrated a statistically significant improvement in cardiorespiratory fitness in favor of the HIIT intervention compared with control conditions (SMD = −0.849; 95% CI: −1.441 to −0.256; *p* = 0.005). The magnitude of the effect was considered large, suggesting that HIIT may substantially improve aerobic capacity in individuals with stroke. However, significant heterogeneity was observed across studies (Q = 39.693; df = 7; *p* < 0.001; I^2^ = 82.37%), indicating considerable variability in the intervention effects. Among the included studies, significant effects favoring HIIT were observed in Boyne et al. [37], Boyne et al. [38], Hsu et al. [42], and Moon [48], whereas Do et al. [39], Kim et al. [43], Gjellesvik et al. [40], and Marzolini et al. [46] did not demonstrate statistically significant between-group differences individually. Despite this variability, the overall pooled effect supported the effectiveness of HIIT for improving cardiorespiratory fitness following stroke (Figure 2).

### 3.6. Meta-Analysis of Cognitive Function

**Figure 3:**
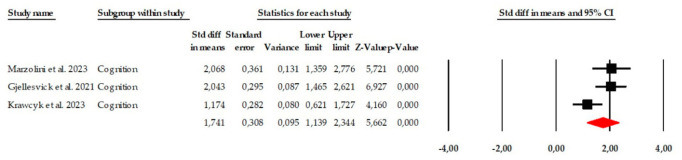
Forest plot of the effects of high-intensity interval training on cognitive function assessed by the Montreal Cognitive Assessment (MoCA) in individuals with stroke. Black squares represent the effect estimates of individual studies, horizontal black lines indicate the corresponding 95% confidence intervals, and the red diamond represents the pooled effect estimate. Arrows indicate the direction of the effect.

Three studies were included in the meta-analysis evaluating cognitive performance using the Montreal Cognitive Assessment (MoCA). The pooled random-effects model demonstrated a statistically significant improvement in cognitive function in favor of HIIT compared with control interventions (SMD = 1.741; 95% CI: 1.139 to 2.344; *p* < 0.001). The magnitude of the pooled effect was large, suggesting that HIIT may substantially improve global cognitive performance in individuals with stroke. Moderate-to-substantial heterogeneity was observed across studies (Q = 5.860; df = 2; *p* = 0.053; I^2^ = 65.87%), indicating some variability across intervention effects. All included studies demonstrated significant individual effects favoring HIIT, including Marzolini et al. [46], Gjellesvik et al. [41], and Krawcyk et al. [45]. Overall, these findings suggest that HIIT may positively influence cognitive recovery following stroke rehabilitation (Figure 3).

### 3.7. Meta-Analysis of Functional Capacity

#### 3.7.1. Gait Speed

**Figure 4:**
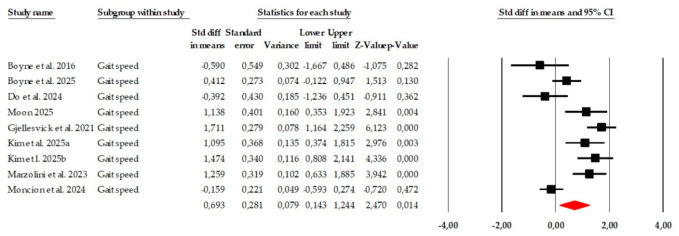
Forest plot of the effects of high-intensity interval training on gait speed (10-Meter Walk Test) in individuals with stroke. Black squares represent the effect estimates of individual studies, horizontal black lines indicate the corresponding 95% confidence intervals, and the red diamond represents the pooled effect estimate. Arrows indicate the direction of the effect.

Nine studies were included in the meta-analysis evaluating gait speed using the 10-Meter Walk Test (10MWT). The pooled random-effects model demonstrated a statistically significant improvement in gait speed in favor of the HIIT intervention compared with control conditions (SMD = 0.693; 95% CI: 0.143 to 1.244; *p* = 0.014). The magnitude of the pooled effect was moderate, suggesting that HIIT may improve walking speed performance in individuals with stroke. However, substantial heterogeneity was identified across studies (Q = 51.834; df = 8; *p* < 0.001; I^2^ = 84.57%), indicating considerable variability in treatment effects. Significant individual effects favoring HIIT were observed in Moon et al. [48], Gjellesvik et al. [41], Kim et al. [43], and Marzolini et al. [46], whereas several studies showed non-significant between-group differences. Despite the observed heterogeneity, the pooled findings suggest that HIIT may be effective for improving gait speed following stroke rehabilitation (Figure 4).

#### 3.7.2. Walking Distance

**Figure 5:**
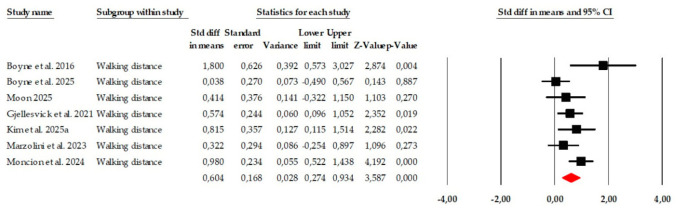
Forest plot of the effects of high-intensity interval training on walking distance assessed by the Six-Minute Walk Test (6MWT) in individuals with stroke. Black squares represent the effect estimates of individual studies, horizontal black lines indicate the corresponding 95% confidence intervals, and the red diamond represents the pooled effect estimate. Arrows indicate the direction of the effect.

Seven studies were included in the meta-analysis assessing walking distance through the Six-Minute Walk Test (6MWT). The pooled random-effects model demonstrated a statistically significant improvement in walking distance in favor of HIIT compared with control interventions (SMD = 0.604; 95% CI: 0.274 to 0.934; *p* < 0.001). The magnitude of the pooled effect was moderate, indicating that HIIT may improve walking endurance and ambulatory capacity in individuals with stroke. Moderate heterogeneity was observed across studies (Q = 12.152; df = 6; *p* = 0.059; I^2^ = 50.63%), suggesting some variability in intervention effects, although heterogeneity remained lower than that observed for gait speed and cardiorespiratory fitness outcomes. Significant individual effects favoring HIIT were identified in Boyne et al. [37], Gjellesvik et al. [41], Kim et al. [44], and Moncion et al. [47], whereas Boyne et al. [38], Moon [48], and Marzolini et al. [46] reported non-significant between-group differences. Overall, these findings suggest that HIIT may be effective for improving walking endurance after stroke rehabilitation (Figure 5).

#### 3.7.3. Functional Mobility

**Figure 6:**
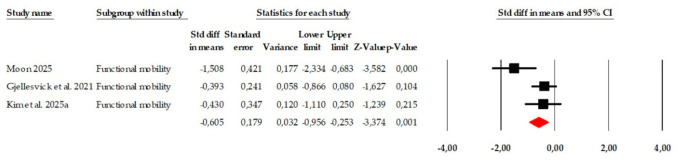
Forest plot of the effects of high-intensity interval training on functional mobility assessed by the Timed Up and Go (TUG) test in individuals with stroke. Black squares represent the effect estimates of individual studies, horizontal black lines indicate the corresponding 95% confidence intervals, and the red diamond represents the pooled effect estimate. Arrows indicate the direction of the effect.

Three studies were included in the meta-analysis evaluating functional mobility using the Timed Up and Go (TUG) test. The pooled random-effects model demonstrated a statistically significant improvement in functional mobility in favor of HIIT compared with control interventions (SMD = −0.711; 95% CI: −1.339 to −0.083; *p* = 0.027). The magnitude of the pooled effect was moderate, indicating that HIIT may improve functional mobility and reduce the time required to complete mobility-related tasks in individuals with stroke. Moderate-to-substantial heterogeneity was observed across studies (Q = 5.629; df = 2; *p* = 0.060; I^2^ = 64.47%), suggesting variability in intervention responses among the included trials. Among the included studies, Moon [48] demonstrated a significant individual effect favoring HIIT, whereas Gjellesvik et al. [41] and Kim et al. [44] did not show statistically significant between-group differences independently. Nevertheless, the pooled analysis supported the beneficial effect of HIIT on functional mobility outcomes following stroke rehabilitation (Figure 6).

#### 3.7.4. Balance

**Figure 7:**
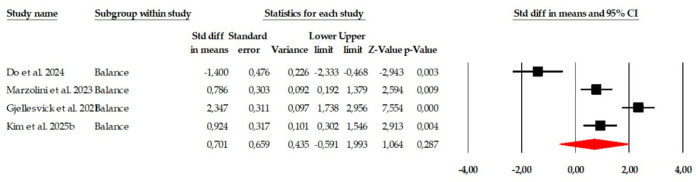
Forest plot of the effects of high-intensity interval training on balance performance assessed by the Berg Balance Scale (BBS) in individuals with stroke. Black squares represent the effect estimates of individual studies, horizontal black lines indicate the corresponding 95% confidence intervals, and the red diamond represents the pooled effect estimate. Arrows indicate the direction of the effect.

Four studies were included in the meta-analysis evaluating balance performance using the Berg Balance Scale (BBS). The pooled random-effects model demonstrated a statistically significant effect in favor of HIIT compared with control interventions (SMD = 2.447; 95% CI: 0.929 to 3.966; *p* = 0.002). The magnitude of the pooled effect was large, suggesting that HIIT may produce substantial improvements in balance performance in individuals with stroke. However, considerable heterogeneity was observed across studies (Q = 43.663; df = 3; *p* < 0.001; I^2^ = 93.13%), indicating marked variability in treatment effects. Individual study effects were generally consistent in favor of HIIT, although the magnitude of improvement varied substantially across studies. These findings suggest that HIIT may improve balance performance after stroke rehabilitation, although the high level of heterogeneity indicates that this result should be interpreted with caution (Figure 7).

### 3.8. Sensitivity Analysis

Sensitivity analyses were conducted using a one-study-removed approach to evaluate the robustness of the pooled findings and to determine whether any individual study disproportionately influenced the overall effect estimates. The results demonstrated that the overall pooled effect remained statistically significant after sequential removal of each study (SMD range ≈ 0.360 to 0.575; *p*-values ranging from 0.002 to 0.041), indicating that no single study substantially altered the overall findings. Although the magnitude of the pooled effect showed minor fluctuations depending on the study excluded, the direction of the effect consistently favored HIIT interventions. These findings reinforce the robustness of the meta-analytic results and suggest that they are not driven by any single trial.

**Figure 8:**
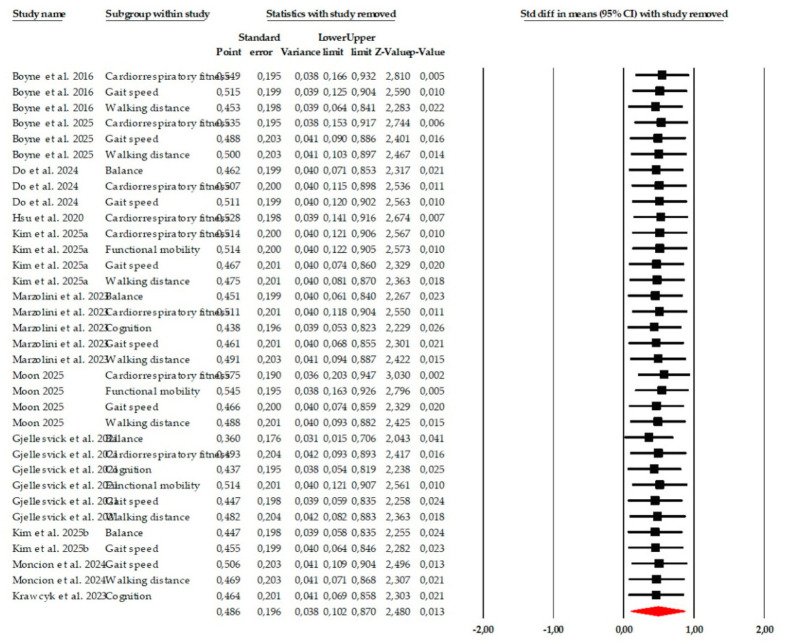
One-study-removed sensitivity analysis assessing the robustness of pooled effects across included studies. Black squares represent the effect estimates of individual studies, horizontal black lines indicate the corresponding 95% confidence intervals, and the red diamond represents the pooled effect estimate. Arrows indicate the direction of the effect.

However, substantial heterogeneity persisted across analyses (I^2^ = 91.63%), suggesting that the observed variability is more likely attributable to differences in intervention protocols, participant characteristics, and outcome measures rather than the influence of an individual outlier study (Figure 8).

## 4. Discussion

This meta-analysis provides an updated synthesis of the evidence on the effects of high-intensity interval training (HIIT) in stroke patients, integrating recent randomized controlled trials that were not considered in previous reviews. The results show significant improvements in cardiorespiratory fitness, cognitive function, and multiple dimensions of functional capacity. However, these findings must be interpreted within the context of a markedly heterogeneous and, at times, contradictory literature, which necessitates a critical analysis beyond statistical significance.

Regarding cardiorespiratory fitness, the significant effect observed in VO_2_peak is consistent with previous reviews that have indicated the potential superiority of HIIT compared to lower-intensity interventions [53,54]. However, these reviews have limitations that the present study addresses more specifically. On the one hand, the review by Baricich et al. [53] focuses primarily on functional outcomes, without systematically integrating the cognitive component, which restricts the understanding of the overall impact of HIIT on post-stroke recovery. On the other hand, the work by Viderman et al. [54] adopts a broad approach that includes multiple clinical populations, which limits the direct applicability of its conclusions to stroke patients. In this sense, the present meta-analysis provides a more specific and clinically relevant synthesis by focusing exclusively on this population and simultaneously integrating cardiorespiratory, functional, and cognitive outcomes. However, the evidence is not entirely consistent. While some included trials report clear improvements, others find no significant differences compared to moderate continuous training, suggesting that HIIT is not inherently superior but highly dependent on the experimental context. In particular, studies employing well-structured active comparators tend to show more attenuated effects, indicating that some of the observed benefit may be related to the total volume of exercise or adherence, rather than intensity per se. This aspect has been highlighted in recent reviews, which question whether the apparent advantage of HIIT is due to specific physiological mechanisms or methodological factors [55]. Beyond the quantitative findings, the systematic review component provides additional insights into the potential mechanisms underlying the observed improvements in cardiorespiratory fitness. Hsu et al. [42] reported that HIIT induced significant increases in serum BDNF levels, oxygen extraction capacity, and peripheral hemodynamic adaptations, suggesting that the benefits of HIIT may extend beyond conventional aerobic conditioning. Similarly, Amanzonwé et al. [36] observed sustained improvements in walking performance and functional outcomes up to six months after the intervention, indicating that some training-induced adaptations may persist over time. These findings, which could not be fully incorporated into the meta-analysis, reinforce the notion that HIIT may promote both physiological and functional recovery through multiple pathways.

Analysis of cognitive function reveals a significant effect favoring HIIT; however, this result should be considered preliminary. The available evidence in this area is limited and based on a small number of studies, which increases the risk of overestimating the effect. Although there are plausible biological rationales, such as increased BDNF, improved cerebral blood flow, and facilitated synaptic plasticity [56], the current literature is inconclusive. In fact, recent reviews have indicated that the effects of exercise on cognition in stroke patients are inconsistent and depend largely on the type of assessment used and the duration of the intervention [30]. Therefore, the results of the present study should be interpreted as indicative, but not definitive. The systematic review also identified several neurophysiological findings that support the potential cognitive benefits of HIIT. Nepveu et al. reported improved motor skill retention and favorable changes in cortical inhibitory mechanisms following high-intensity exercise, whereas Rodrigues et al. observed modifications in corticospinal excitability and interhemispheric balance after training. Although these outcomes were not included in the quantitative synthesis due to methodological differences, they provide mechanistic evidence suggesting that HIIT may facilitate neuroplasticity and neural recovery after stroke. Nevertheless, the limited number of available studies and the diversity of cognitive and neurophysiological assessment methods warrant cautious interpretation.

In terms of functional capacity, the results of this study show consistent but moderate effects on variables such as gait speed, distance covered, and functional mobility, along with a significant effect on balance. However, these results should be interpreted with caution. Previous literature has shown notable inconsistency in these outcomes, especially in variables such as the 10MWT or TUG, where some studies report significant improvements while others find no differences between groups [29]. This discrepancy suggests that the impact of HIIT on locomotor function is neither direct nor universal, but rather mediated by factors such as training specificity, the inclusion of functional tasks, and the participants’ baseline fitness level. In this regard, protocols that combine HIIT with task-oriented training or gait-specific rehabilitation appear to generate greater benefits, which challenges the idea that intensity alone is the main determinant of functional improvement. Findings from the systematic review further suggest that functional improvements may depend on the specific characteristics of the intervention. For example, studies combining HIIT with robot-assisted gait training or task-oriented rehabilitation generally reported greater gains in walking performance, balance, and lower-limb function than studies using HIIT as a standalone intervention. In addition, Pressler et al. demonstrated improvements in gait symmetry parameters, highlighting that HIIT may positively influence movement quality as well as functional performance. These observations support the hypothesis that the effectiveness of HIIT may be enhanced when integrated with rehabilitation approaches specifically targeting locomotor recovery.

A key finding that reinforces the critical interpretation of the results is the high degree of heterogeneity observed in most of the analyses. This aspect cannot be considered a secondary element, but rather central to the interpretation of the effects. The heterogeneity reflects not only methodological differences, but also a lack of standardization in the very definition of HIIT in the context of post-stroke rehabilitation. The included protocols vary widely in terms of relative intensity, interval duration, exercise modality, and total intervention duration. This variability limits the ability to draw generalizable conclusions and suggests that the term “HIIT” is being used as a broad and nonspecific construct. The sensitivity analysis performed confirms that the results are not driven by individual studies, but it does not resolve the fundamental issue of structural inconsistency between studies.

In terms of clinical applicability, although HIIT has been described as a safe and feasible intervention in clinical populations [57], its implementation in the real-world context of neurological rehabilitation presents significant challenges. The need for specialized supervision, monitoring of intensity, and variability in exercise tolerance limit its universal applicability. Furthermore, most of the included studies have been conducted in relatively functional patients, which introduces selection bias and restricts the generalizability of the results to patients with greater disability.

This study has significant strengths, such as the exclusive inclusion of randomized controlled trials, the assessment of multiple clinically relevant domains, and the incorporation of sensitivity analyses. However, these strengths do not eliminate inherent limitations of the available evidence. These include methodological heterogeneity, the small number of studies for some outcomes (especially cognition), and the inability to control for key variables such as actual adherence to training or the intensity achieved. Furthermore, the lack of blinding in exercise interventions remains a potential source of bias.

Overall, the results of this meta-analysis suggest that HIIT is a promising, but not definitive, intervention in post-stroke rehabilitation. Rather than confirming its superiority, current evidence indicates that its effectiveness depends on multiple contextual and methodological factors. Therefore, future research should focus on standardizing protocols, identifying subgroups of patients who benefit most, and evaluating long-term outcomes. Furthermore, it is necessary to move towards experimental designs that allow the isolation of the specific effect of intensity from other components of training.

## 5. Conclusions

This systematic review and meta-analysis suggest that high-intensity interval training (HIIT) is a promising rehabilitation strategy for individuals with stroke, with potential benefits for cardiorespiratory fitness, cognitive function, and multiple dimensions of functional capacity. Beyond the quantitative improvements identified in the meta-analysis, the systematic review revealed additional evidence of neurophysiological and functional adaptations, including enhanced neuroplasticity-related mechanisms, improvements in motor learning, gait symmetry, and cardiovascular responses. However, the available evidence remains characterized by substantial methodological and clinical heterogeneity, differences in intervention protocols, and a limited number of studies for certain outcomes, particularly cognition. Therefore, while HIIT appears to be a feasible and potentially effective intervention in post-stroke rehabilitation, its effectiveness may depend on patient characteristics, intervention design, and rehabilitation context. Future research should focus on standardizing HIIT protocols, identifying the patient subgroups most likely to benefit, and evaluating long-term clinical and neurophysiological outcomes through adequately powered randomized controlled trials.

## Figures and Tables

**Table 1:** Methodological quality of the included articles.

	1 *	2	3	4	5	6	7	8	9	10	11	Total Score
Amanzonwé et al. 2025 [36]	1	1	1	1	1	0	1	1	1	1	1	9
Boyne et al. 2016 [37]	1	1	1	0	0	0	1	1	1	1	1	7
Boyne et al. 2025 [38]	1	1	1	1	0	0	1	1	1	1	1	8
Do et al. 2021 [39]	1	1	1	1	0	0	1	1	1	1	1	8
Gjellesvik et al. 2020 [40]	1	1	1	1	0	0	1	0	1	1	1	7
Gjellesvik et al. 2021 [41]	1	1	1	1	0	0	1	0	1	1	1	7
Hsu et al. 2021 [42]	1	1	1	1	0	0	1	1	1	1	1	8
Kim et al. 2025 [43]	1	1	1	1	0	0	0	1	1	1	1	7
Kim et al. 2025 [44]	1	1	1	1	0	0	1	1	1	1	1	8
Krawcyk et al. 2023 [45]	1	1	1	1	0	0	1	0	1	1	1	7
Marzolini et al. 2023 [46]	1	1	1	1	0	0	1	1	1	1	1	8
Moncion et al. 2024 [47]	1	1	1	1	0	0	1	1	1	1	1	8
Moon 2025 [48]	1	1	1	1	0	0	1	1	1	1	1	8
Nepveu et al. 2017 [49]	1	1	0	1	0	0	1	1	1	1	1	7
Pressler et al. 2025 [50]	1	1	0	1	0	0	1	1	1	1	1	7
Rodrigues et al. 2025 [51]	1	1	1	1	0	0	0	1	1	1	1	7
Rodrigues et al. 2025 [52]	1	1	1	1	0	0	0	1	1	1	1	7

Items: 1: eligibility criteria; 2: random allocation; 3: concealed allocation; 4: baseline comparability; 5: blind subjects; 6: blind therapists; 7: blind assessors; 8: adequate follow-up; 9: intention-to-treat analysis; 10: between-group comparisons; 11: point estimates and variability; yes = 1; no = 0. * Item 1 was not included in the calculation of the total score.

**Table 2:** RoB-2 to assess the risk of bias.

BIAS	Bias in Randomization	Bias Due to Deviations from the Intervention.	Bias Due to Missing Data	Bias in the Measurement of Outcomes	Bias in the Selection of Reports	Overall Assessment of the Risk Bias
Amanzonwé et al. 2025 [36]	Low risk	Low risk	Low risk	Low risk	Low risk	Low risk
Boyne et al. 2016 [37]	Low risk	Unclear risk	Low risk	Low risk	Low risk	Low or unclear risk
Boyne et al. 2025 [38]	Low risk	Low risk	Low risk	Low risk	Low risk	Low risk
Do et al. 2021 [39]	Low risk	Low risk	Low risk	Low risk	Low risk	Low risk
Gjellesvik et al. 2020 [40]	Low risk	Low risk	Low risk	Low risk	Low risk	Low risk
Gjellesvik et al. 2021 [41]	Low risk	Low risk	Low risk	Low risk	Low risk	Low risk
Hsu et al. 2021 [42]	Low risk	Low risk	Unclear risk	Low risk	Low risk	Low or unclear risk
Kim et al. 2025 [43]	Low risk	High risk	Low risk	High risk	Low risk	Unclear risk
Kim et al. 2025 [44]	Low risk	Low risk	Low risk	Low risk	Low risk	Low risk
Krawcyk et al. 2023 [45]	Low risk	Low risk	Low risk	Low risk	Low risk	Low risk
Marzolini et al. 2023 [46]	Low risk	Low risk	Low risk	Unclear risk	Low risk	Low or unclear risk
Moncion et al. 2024 [47]	Low risk	Low risk	Low risk	Unclear risk	Low risk	Low or unclear risk
Moon 2025 [48]	Low risk	Low risk	Low risk	Low risk	Low risk	Low risk
Nepveu et al. 2017 [49]	Low risk	Low risk	Low risk	Unclear risk	Low risk	Low or unclear risk
Pressler et al. 2025 [50]	Low risk	Low risk	Unclear risk	Low risk	Low risk	Low or unclear risk
Rodrigues et al. 2025 [51]	Low risk	Low risk	Unclear risk	Unclear risk	Low risk	Low or unclear risk
Rodrigues et al. 2025 [52]	Low risk	Low risk	Unclear risk	Unclear risk	Low risk	Low or unclear risk

**Table 3:** Characteristics of the included studies.

Author and Year		Sample CG/IG	Control Group	Intervention Group			
	Sex			Age	Treatment	Exercise Parameters	Results
Amanzonwé et al. 2025 [36]	F: 45.5% M: 54.5%	22/22	combining unloaded cycling (SHAM) to conventional physiotherapy	56.8	HIIT cycling	F: 3 times/week #S: 18 sessions D: 30 min	The 2-way factorial analysis of variance showed a significant interaction of time × group on WRpeak, 6MWT, 10mWT, and mRS. The significant interaction indicates that the change in WRpeak, 6MWT, 10mWT, and mRS after 6-week of training was significantly greater for HIIT cycling versus SHAM. These changes are also significantly greater in the HIIT group vs. the SHAM group up to 6 months post-training.
Boyne et al. 2016 [37]	F: 48.3% M: 51.7%	5/11	MICT	58	HIIT	F: 3 times/week #S: 12 sessions D: 25 min	During the 8-month recruitment period, 26 participants consented to participate. Eighteen participants were enrolled and randomly assigned to either the HIIT group or the MICT group. Eleven out of the 13 HIIT group participants attended all sessions. Participants reported that HIT was acceptable and no serious adverse events occurred. Standardized effect size estimates between groups were moderate to very large for most outcome measures. Only 30% of treadmill speed gains in the HIIT group translated into overground gait speed improvement.
Boyne et al. 2025 [38]	F: 34.7% M: 65.3%	28/27	MAT	62.7	HIIT	F: 3 times/week #S: 36 sessions D: 45 min	Net gains in 6MWD from HIIT versus MAT were primarily mediated by faster training speeds and longitudinal gait adaptations. Training step count was also positively associated with 6MWD gains but was lower with HIIT versus MAT, which decreased the net 6MWD gain from HIIT. HIIT generated higher training heart rate and lactate than MAT, but aerobic capacity gains were similar between groups, and 6MWD changes were not associated with training heart rate, training lactate, or aerobic adaptations.
Do et al. 2021 [39]	F: 31.8% M: 68.2%	11/11	Home exercise education including aerobic, balance, stretching and strengthening exercises	62.7	RAGT	F: 3 times/week #S: 24 sessions D: 20 min	RAGT significantly improved VO_2_max, gait, balance, and lower limb strength compared with controls, with significant improvements in 2MWT test, 10MWT, Motricity Index-Lower, and Fugl-Meyer Assessment outcomes. No changes were seen in muscle mass or blood markers.
Gjellesvik et al. 2020 [40]	F: 42% M: 58%	34/36	Standard care	57.6	HIIT treadmill training	F: 3 times/week #S: 24 sessions D: 30 min	Mean baseline VO_2_peak was 2.63 ± 1.08 L·min^−1^ vs. 2.87 ± 0.71 L·min^−1^, while at 12 months VO_2_peak was 2.70 ± 1.00 L·min^−1^ vs. 2.67 ± 0.76 L·min^−1^ in the intervention and control groups, respectively. There was a significant and greater improvement in the intervention group compared with the control group at 12 months in 3 of 6 secondary outcomes from the peak test but no significant differences for blood pressure or blood profile.
Gjellesvik et al. 2021 [41]	F: 41.5% M: 58.5%	34/36	Standard care	58.2	Treadmill HIIT	F: 3 times/week #S: 24 sessions D: 30 min	The intervention group showed a significant treatment effect from baseline to posttest on a 6 min walk test of 28.3 m; Berg Balance Scale 1.27 points; and Trail Making Test Part B. The intervention group showed significantly greater improvement on TMT-B at the 12-month follow-up. The control group showed significantly greater improvement in total Functional Independence Measure score with a treatment effect of 2.37 points at 12-month follow-up. No significant differences were identified between groups on other outcomes at any time point.
Hsu et al. 2021 [42]	F: 13% M: 87%	13/10	MICT	58.3	HIIT	F: 3 times/week #S: 36 sessions D: 30 min	HIIT induced significant increases in VO_2_peak, CO, Δ[HHb], Δ[THb], and serum BDNF level. The improvement in VO_2_peak was significantly greater with HIIT than MICT, as was AV O2diff, Δ[HHb], and serum BDNF level. HIIT facilitated neuron dendritic protrusions with prominent redistribution of mitochondria.
Kim et al. 2025 [43]	F: 38.9% M: 61.1%	22/22	Treadmill-based gait therapy	61.6	RAGT and HIIT	F: 3 times/week #S: 24 sessions D: 30 min	Between-group comparisons showed significant improvements in the intervention group in 10MWT, FAC, BBS, 2MWT, and FMA-LE scores. Additionally, the intervention group demonstrated enhanced 2MWT and VO_2_max within group; however, lean body mass within-group changes were minor in both groups. The superior outcomes in the intervention group highlight the potential combined benefits of combining HIIT with RAGT for intensive, repetitive, and task-specific training.
Kim et al. 2025 [44]	F: 35.3% M: 64.7%	17/17	HIIT treadmill training	54.7	Task-oriented treadmill training with HIIT	F: 3 times/week #S: 12 sessions D: 30 min	There was an interaction effect between time and group for 10MWT, FGA, 6MWT, and TUG. Post hoc tests revealed that 10MWT, FGA, and 6MWT showed significantly greater improvements in the EG compared to the CG, and in the intragroup comparison, both EG and CG showed significant improvements over time. The TUG showed no significant difference between groups, and within-group comparisons showed that both EG and CG had a significant decrease over time.
Krawcyk et al. 2023 [45]	F: 18.5% M: 81.5%	31/28	Usual care	63.9	HIIT including weekly motivational calls	F: 5 times/week #S: 60 sessions D: 15 min	No change was detected in cardiorespiratory fitness between groups from baseline to 12-month follow-up. At six months, vigorous-intensity activity was maintained in the intervention group and increased in the usual care group, with no difference between groups. Vigorous-intensity activity declined to baseline levels at 12 months in both groups. Secondary outcomes improved from baseline to 12 months with no significant differences between groups. Similar rate of recurrent stroke occurred in each group with a three-month delay in the intervention group.
Marzolini et al. 2023 [46]	F: 19% M: 81%	23/24	MICT	62	HIIT	F: 3 times/week #S: 72 sessions D: 20 min	Main outcomes were change in VO_2_peak and 6MWT. Assessors were blinded to the treatment group for VO_2_peak but not 6MWD. Secondary outcomes were change in ventilatory anaerobic threshold, cognition, gait economy, 10 m gait velocity, balance, stair-climb performance, strength, and quality of life. Change in VO_2_peak for MICT versus HIIT was 2.4 ± 2.7 versus 5.7 ± 3.1 mL·kg^−1^·min^−1^, and change in 6MWD was 70.9 ± 44.3 versus 83.4 ± 53.6 m. HIIT had greater improvement in ventilatory anaerobic threshold. No other between-group differences were observed.
Moncion et al. 2024 [47]	F: 39% M: 61%	40/42	MICT	64.9	HIIT	F: 3 times/week #S: 36 sessions D: 20 min	A significant group × study time point interaction was found for VO_2_peak at 12 weeks whereby the HIIT group had greater gains in VO_2_peak compared with the MICT group. There was no between-group difference in VO_2_peak at 8-week follow-up. No group × study time point interactions were found for cardiovascular risk factors or mobility outcomes.
Moon 2025 [48]	F: % M: %	14/15	MICT	51.3	HIIT and conventional physical therapy	F: 3 times/week #S: 18 sessions D: 70 min	In this higher-functioning cohort, HIIT showed significantly greater improvements than MICT in VO_2_max, HRmax, walking heart rate, 10MWT, TUG test, and 6MWT. Lipid profiles improved significantly within the HIIT group only, and no between-group differences were observed.
Nepveu et al. 2017 [49]	F: 27.3% M: 72.7%	11/11	Non exercise control group	64.9	HIIT	F: 3 times/week #S: 3 sessions D: 15 min	The graded exercise test reduced interhemispheric imbalances in GABAa-mediated short-interval intracortical inhibition but changes in other markers of excitability were not statistically significant. The group that performed high-intensity interval training showed better retention of the motor skill.
Pressler et al. 2025 [50]	F: 34.7% M: 65.3%	28/27	MAT	62.7	HIIT	F: 3 times/week #S: 36 sessions D: 45 min	Non-paretic step length increased significantly more with HIIT compared to MAT. Both groups demonstrated significant increases in cadence and bilateral single support time, and decreases in the coefficient of variation for stride velocity, time, and length. Changes in step length symmetry were only apparent when assessing individuals with baseline asymmetry.
Rodrigues et al. 2025 [51]	F: 35.5% M: 64.5%	28/28	MICT	66.4	HIIT	F: 3 times/week #S: 36 sessions D: 24 min	CSE changes were not significantly different between HIIT and MICT but exploratory analyses showed that, when analyzed together, both groups increased resting MEP amplitude, decreased rMT, and reduced ICF in the ILH. No CSE changes in the CLH were observed. HIIT and MICT rebalanced interhemispheric rMT and ICF ratios, and increased resting MEP amplitude ratio.
Rodrigues et al. 2025 [52]	F: 38% M: 62%	34/47	MICT	65.5	HIIT	F: 3 times/week #S: 36 sessions D: 24 min	HIIT elicited a lower effective response, that also progressively declined during sessions, in contrast to MICT. HIIT and MICT did not elicit any significant difference between groups or change over time for post-exercise enjoyment or any motivation constructs.

HIIT: High-Intensity Interval Training; WRpeak: Work Rate peak; 6MWT: 6-Minute Walk Test; 10MWT: 10-Meter Walk Test; mRS: modified Rankin Scale; MICT: Moderate-Intensity Continuous Aerobic Training; MAT: Moderate-intensity Aerobic Training; RAGT: Robot-Assisted Gait Rehabilitation; VO_2_peak: peak oxygen consumption; BDNF: Brain Derived Neurotrophic Factor; Δ[HHb]: deoxyhemoglobin; Δ[THb]: total hemoglobin; AV O2diff: arteriovenous oxygen difference; FAC: Functional Ambulation Category; BBS: Berg Balance Scale; 2MWT: 2-Minute Walk Test; FMA-LE: Fugl-Meyer Assessment–Lower Extremity; FGA: Functional Gait Assessment; TUG: Timed Up and Go; EG: Experimental Group; CG: Control Group; HRmax: maximum Heart Rate; GABAa: γ-aminobutyric acid; CSE: Corticospinal Excitability; MEP: Motor Evoked Potential; rMT: resting Motor Threshold; ICF: Intracortical Facilitation; CLH: Contralesional Hemispheres.

## Data Availability

No new data were created or analyzed in this study.

## Supplementary Materials

The following supporting information can be downloaded at: https://www.mdpi.com/article/10.3390/jcm15134977/s1, Table S1: PRISMA 2020 checklist.

## Abbreviations

The following abbreviations are used in this manuscript:

HIIT	High-Intensity Interval Training
MICT	Moderate-Intensity Continuous Training
VO_2_peak	Peak Oxygen Consumption
VO_2_max	Maximal Oxygen Consumption
RCT	Randomized Controlled Trial
SMD	Standardized Mean Difference
CI	Confidence Interval
BBS	Berg Balance Scale
TUG	Timed Up and Go
6MWT	Six-Minute Walk Test
10MWT	Ten-Meter Walk Test
MoCA	Montreal Cognitive Assessment
BDNF	Brain-Derived Neurotrophic Factor
PEDro	Physiotherapy Evidence Database
RoB-2	Risk of Bias 2 Tool
